# Green bean biofortification for Si through soilless cultivation: plant response and Si bioaccessibility in pods

**DOI:** 10.1038/srep31662

**Published:** 2016-08-17

**Authors:** Francesco Fabiano Montesano, Massimiliano D’Imperio, Angelo Parente, Angela Cardinali, Massimiliano Renna, Francesco Serio

**Affiliations:** 1 Institute of Sciences of Food Production, CNR – National Research Council of Italy, Via G. Amendola, 122/O–70126 Bari, Italy; 2 Department of Agricultural and Environmental Science – University of Bari Aldo Moro, Via Amendola, 165/A–70126 Bari, Italy

## Abstract

Food plants biofortification for micronutrients is a tool for the nutritional value improvement of food. Soilless cultivation systems, with the optimal control of plant nutrition, represent a potential effective technique to increase the beneficial element content in plant tissues. Silicon (Si), which proper intake is recently recommended for its beneficial effects on bone health, presents good absorption in intestinal tract from green bean, a high-value vegetable crop. In this study we aimed to obtain Si biofortified green bean pods by using a Si-enriched nutrient solution in soilless system conditions, and to assess the influence of boiling and steaming cooking methods on Si content, color parameters and Si bioaccessibility (by using an *in vitro* digestion process) of pods. The Si concentration of pods was almost tripled as a result of the biofortification process, while the overall crop performance was not negatively influenced. The Si content of biofortified pods was higher than unbiofortified also after cooking, despite the cooking method used. Silicon bioaccessibility in cooked pods was more than tripled as a result of biofortification, while the process did not affect the visual quality of the product. Our results demonstrated that soilless cultivation can be successfully used for green bean Si biofortification.

Plant biofortification for micronutrients is arising increasing interest as a tool to improve the nutritional value of food plants, in the framework of the current worldwide challenge to produce, in a sustainable way, more and better food^1^. Two basic approaches can be adopted for biofortification: i) increase of nutrient (micro and/or macronutrient) content in food plants^2^,^3^; ii) reduction of antinutritional factors, such as phytates and oxalates (compounds able to reduce the bioavailability of nutrients)^4^. Both approaches can be achieved through different mechanisms, such as conventional plant breeding, genetic engineering and agronomic procedures^4^.

Silicon (Si) is a general mineral component present in many food plants, including cereals, fruit, vegetables and legumes^5^. The Si content in plant tissues is generally related with species^6^,^7^. Its absorption in the intestinal tract is related to the food source. As an example, it is well absorbed from alcohol-free beer (64% of dose) and green beans (44%); in contrast, it is poorly absorbed (4%) from bananas^5^. Regarding nutritional aspects, Si intake is positively associated with promotion of bone formation, increase of bone mineral density in men and promenopausal women^8^,^9^,^10^.

Several *in vitro* studies were performed in order to determine the plausible Si mechanism of action on bone^11^. Authors reported that Si, in *in vitro* studies, improved different parameters, such as cell proliferation, alkaline phosphatase activity and osteocalcin, and that Si increases cell type I collagen, in the osteosarcoma cell line MG-63 and the osteoblastic cell line HCC1^8^,^12^. Recent *in vivo* and epidemiological evidences indicate that nutritional intakes of Si are beneficial for bone health in humans^9^,^13^. Nielsen (2006)^14^ suggests that an adequate intake, able to improve of bone health, might be between 10 and 25 mg∙day^−1^.

Although Si is generally not considered to be essential for higher plants^15^, its availability very significantly improves plant fitness in nature and increases agricultural productivity^16^,^17^. Under an horticultural point of view, previous research focused on the role of Si in inducing protection against biotic stress^18^, and in alleviating the effects of salt stress^19^. In green bean (*Phaseolus vulgaris* L.) the Si improved the physiological condition under salinity stress by reducing the tissue Na content^20^.

On the other hand, it has been recently demonstrated that it is possible to increase the Si content and Si bioaccessibility (the percentage of bioactive compounds released from the food matrix during the digestion process), and as a result the nutritional value, in food plants by using biofortification process^6^. In this previous study, performed by the same team of the present research, soilless cultivation (floating system, a typical hydroculture technique), allowing optimal Si fertilization control and mineral plant uptake, was used for the Si biofortification of six leafy vegetables, frequently used as fresh-cut products, resulting in increased Si plant tissue content and bioaccessibility^6^.

The green bean is one of the most important crops in the world and it is widely cultivated in the Mediterranean basin in greenhouse conditions. Although still a relatively understudied crop in comparison with other greenhouse crops, green bean cultivation on soilless substrates is arising lively interest because of the potential to produce high value income^21^. In fact, a relevant part of greenhouse vegetables, especially in Europe, U.S. and Canada, are produced using soilless cultivation systems based on horticultural growing media. The main advantages of soilless over traditional cultivation systems consist in that nutrients, oxygen and water required for a healthy plant growth are better controlled, and that soil-borne diseases can be prevented, generally resulting in increased yield and high quality^22^,^23^,^24^. A more efficient use of resources, especially water and fertilizers, can also be achieved in soilless cultivation systems^25^.

Starting from the above mentioned considerations, the green bean could be considered a good target for Si biofortification. The aims of this study were: i) to evaluate the possibility to obtain Si-enriched green bean pods by acting on the fertilizer solution composition in typical soilless system conditions; ii) to assess the influence of different cooking methods (boiling and steaming) on Si content, visual quality (color parameters) and Si bioaccessibility (by using an *in vitro* gastro-intestinal digestion process) of green bean pods.

We hypothesized that soilless cultivation on substrate, offering the possibility to modify *ad hoc* the mineral nutrition of plants, could be used as an effective tool for the Si biofortification of green bean pods without affecting negatively the overall crop performance (in terms of yield parameters). Since green bean is a vegetable typically consumed after cooking, we were also interested in verify that the cooking has no negative effects on Si content and bioaccessibility of Si-enriched pods.

## Materials and Methods

### Experimental conditions

The experiment was carried out from May to July 2015 in a plastic (polymethacrylate) greenhouse at the Experimental Farm “La Noria” of the Institute of Sciences of Food Production (ISPA-CNR) in Mola di Bari (BA), southern Italy (41°03′ N, 17°04′ E).

Green bean (*Phaseolus vulgaris* L.) cultivar ‘Saporro’, one of the most widely-grown varieties in Mediterranean conditions due to its high productivity and satisfactory fruit quality, was used in the experiment. The seedlings, obtained from a commercial nursery, were transplanted on May 5^th^ 2015, two per pot, at the second true leaf stage into 9.5 L PVC pots, containing 1–2 mm grain-size perlite [Agrilit P2, Perlite Italiana, Corsico (MI), Italy]. Pots were placed on troughs 1.30 m apart, covered with polyethylene film and placed on 1.5% slope.

The growing medium was saturated with water before transplanting. Before the start of treatments, plants were watered via a drip irrigation system with a nutrient solution (NS) prepared with pre-collected rain water [0.15 mM Ca, 0.04 mM K, 0.02 mM Mg, 0.21 mM Na, Si below the detection limit (see Table S1 in supplementary information), EC = 0.06 dS∙m^−1^, pH = 7.4] and containing 13.8 mM N, 5.5 mM K, 1.3 mM P, 1.2 mM Mg, 3.7 mM Ca, 1.8 mM S, with micronutrients applied according to Johnson *et al*.^26^. The Si concentration in this NS was 0.073 mM, as a result of traces in the fertilizer salts used. The NS had an EC of ≈1.9 dS∙m^−1^ and pH was adjusted to 5.5. All plants were well-watered using the unique above mentioned NS for 27 days after transplanting (DAT) to allow the seedlings to establish, then treatments were started. Treatments consisted in the ‘Unbiofortified’, in which plants were irrigated with the above described NS up to the end of the experiment, and the ‘Biofortified’, in which a NS containing 3.6 mM Si was used. The target Si concentration was reached by adding potassium metasilicate (K_2_SiO_3_) to the NS. Potassium added by K_2_SiO_3_ was taken into account in the formulation of the nutrient solutions and the additional K introduced as K_2_SiO_3_ was balanced acting on K_2_SO_4_ dose in the ‘Unbiofortified’ treatment^6^. Irrigation was automatically operated by a programmable electronic timer according to a predetermined schedule set in order to prevent any drought stress event. Schedule was adjusted based on increasing plant size and water needs.

Average daily mean, minimum and maximum air temperatures inside the greenhouse over the experiment were 26.3, 18.6 and 35.6 °C, respectively. Average daily mean, minimum and maximum air relative humidity values were 58, 35 and 81%, respectively. The daily light integral (DLI) during the experiment ranged from about 7 to 26, with a mean value of 22 mol∙m^−2^∙d^−1^. The growing cycle was terminated on July 9^th^ 2015, 56 DAT. Plants were arranged in a randomized complete block design with three replications. The single experimental unit/replication was represented by a trough containing 22 plants.

### Measurements and Analysis

#### Yield determination and sampling procedures

Harvests started 36 DAT, when the first pods were visually considered suitable for the market standard typical for this variety (pod length between 12–14 cm). Harvests were carried out approximately every 3–4 days up to the end of the experiment, when plants ended their pod production. At each harvest, the pods were counted and weighted, and a representative sample from each experimental unit was used to determine the dry matter percentage of the pods, by oven-drying the fresh material at 65 °C in a thermo-ventilated oven, up to constant weight. Samples of pods harvested on 36, 41 and 56 DAT were analyzed in order to determine the Si concentration (see after for a detailed description of the analysis procedure). At the end of the growing cycle, representative samples of leaves, stems and pods were obtained from each experimental unit. The material was oven dried at 65 °C and then analyzed in order to determine the Si concentration in the different aerial organs of the plant.

Pods harvested on 41 DAT were subjected to two different cooking methods and then analyzed in order to determine the Si concentration and the color parameters; the assessment of Si bioaccessibility in biofortified and unbiofortified cooked pods was determined (see after for a detailed description of the analysis procedure).

#### Silicon content analysis

For the determination of Si in vegetal material samples (raw and cooked pods, leaves and stems), 1 g of dried sample was ashed in a muffle furnace at 550 °C and digested with 20 mL of 1 mol∙L^−1^ HCl in boiling H_2_O (99.5 ± 0.5 °C) for 30 min. The resulting solution was filtered, diluted and analyzed by spectroscopy method (ASTM Method D859-00.27)^27^ for the determination of Si content as reported in D’Imperio *et al*.^6^. Briefly, the solutions required for this procedure were (NH_4_)_6_Mo_7_O_24_·4H_2_O (75 g∙L^−1^) and HO_2_C_2_O_2_H (10 g∙L^−1^). A 1:1 HCl:H_2_O solution and a reducing solution were also used. The reducing solution was composed of 30 g of NaHSO_3_, 1.5 g of Na_2_SO_3_, and 0.5 g of H_2_NC_10_H_5_(OH)SO_3_H dissolved in 200 mL in H_2_O. An aliquot of sample (including blanks) or standard, 1 mL of the HCl solution and 2 mL of (NH_4_)_6_Mo_7_O_24_·4H_2_O solution were added in rapid succession. The solution was mixed and allowed to sit for 5 min. After 5 min, 1.5 mL of oxalic acid were added and allowed to sit for 1 min. Two milliliters of the reducing solution were added, and the solution was allowed to sit for 10 min. The absorbance of the samples was determined at 640 nm. The standards for Si analysis were made from a 25 mg∙L^−1^ Si stock solution (prepared in MilliQ water), standard concentrations ranged from 0.40 to 5.68 mg∙L^−1^. The quantification of Si in vegetables was determined by interpolation with a calibration curve, previously made with an R^2^ = 0.9997. Quality assurance (QA) data are reported in Table S1.

#### Cooking procedure

After removing inedible parts with a knife, green bean pods were washed with deionized water. Two cooking treatments (boiling and steaming) were applied both for unbiofortified and biofortified pods. Three replicates for each application (about 300 g of pods) were prepared in order to provide independent replicates for each cooking method and for the uncooked samples. Therefore, a total of 18 samples were prepared: 2 growing treatments (biofortified and unbiofortified) x 3 post-harvest treatments (raw, boiling and steaming) x 3 replicates. In order to obtain samples for chemical analysis, a half portion of each replicate was freeze-dried and ground to powder. The samples were then packed in hermetic jars and stored in the dark at −21.0 ± 1.0 °C until the analyses were carried out, as described in the sections below. Meanwhile, the remaining half portions of each replicate were used for color analysis, carried out as described in the sections below. The cooking treatments were as follows:

Boiling: pods were boiled in a steel pot with boiling distilled water (99.0 ± 1.0 °C) for 6 min at a pods/water ratio of 1:6. The samples were drained off and rapidly cooled on ice.

Steaming: pods were placed on a tray in a steam cooker (VC 101 630 Tefal, Italy) covered with a lid and cooked with water vapour (99.0 ± 1.0 °C) for 10 min under atmospheric pressure. The samples were rapidly cooled on ice.

#### Color analysis

Color analysis of raw and cooked pods was conducted with a colorimeter (CR-400, Konica Minolta, Osaka, Japan) equipped with illuminant D65, in reflectance mode and in the CIE L^*^ (lightness), a^*^(redness) b^*^ (yellowness) color scale. For each replicate, color parameters were measured at ten random points on the peel surface of ten pods. Before the measurements, the colorimeter was calibrated with a standard reference having values of L^*^, a^*^ and b^*^ corresponding to 97.05, 0.11 and 1.79, respectively. Hue angle (h° = arctg b^*^/a^*^) and saturation (Chroma = [a^*2^ + b^*2^]^1/2^) were then calculated from primary L^*^, a^*^ and b^*^ readings.

#### Assessment of silicon bioaccessibility in biofortified and unbiofortified cooked pods

The *in vitro* digestion model was carried out as described by Ferruzzi *et al*.^28^. Samples of cooked green bean pods (boiling and steaming) were homogenized with 0.9% NaCl at the initial pH of 7. The gastric phase was initiated with addition of pepsin solution (40 mg∙mL^−1^ in 0.1 M HCl) and adjustment of pH to 2.5 ± 0.1. Samples were flushed with N_2_ (in order to reduce oxidation of compounds) and incubated at 37 °C and mixed for one hour by head-over-heels (1 × g at 37 °C) (Rotator Type L2, Labinco BV, Breda, The Netherlands). Following gastric digestion, the small intestinal phase was initiated by adjusting the pH of gastric digesta to 5.3 with a combination of NaHCO_3_ (100 mM) and NaOH (1 M) followed by the addition of small intestinal enzyme solution porcine lipase (2 mg∙mL^−1^ in 100 mM NaHCO_3_), pancreatin (4 mg∙mL^−1^ in 100 mM NaHCO_3_) and bile (24 mg∙mL^−1^in 100 mM NaHCO_3_). The final sample pH was adjusted to 6.5 ± 0.1; the volume was standardized to 15 mL with solution (0.9% NaCl) and sample blanketed with N_2_, incubated at 37 °C and mixed for two hours by head-over-heels (1 × g at 37 °C). The samples were centrifuged at 10000 × g for 1 hour at 4 °C to separate the aqueous intestinal digesta (AQ) from the residual solid. Aliquots of undigested AQ were collected, filtered using a 0.2 μm PTFE filter and stored frozen at −80 °C under a blanket of nitrogen until analysis by a spectroscopy method for the determination of Si content. As regards Si content in digested fluid was determined by using the protocol described above, with the difference of the spectrophotometric readings at 815 nm. Blank correction was performed and subtracted in all analyses, in order to reset the contribution of blanks due to trace elements, Si included, normally present in bioaccessibility reagents^29^. From a Si stock solution of 1.43 mg∙L^−1^, the Si analysis was performed by using standard concentrations ranging from 1402 to 352 μg∙L^−1^ of Si. The quantification of Si in digested fluids was determined by interpolation of a calibration curve with an R^2^ = 0.9994. Quality assurance (QA) data are reported in Table S1.

#### Statistical analysis

The experimental data were subjected to analysis of variance (ANOVA). Treatment means were separated by LSD test when there was a significant difference at the P < 0.05 level. The statistical software STATISTICA 10.0 (StatSoft, Tulsa, OK, USA) was used for the analysis.

## Results

### Crop performance

**Figure 1:**
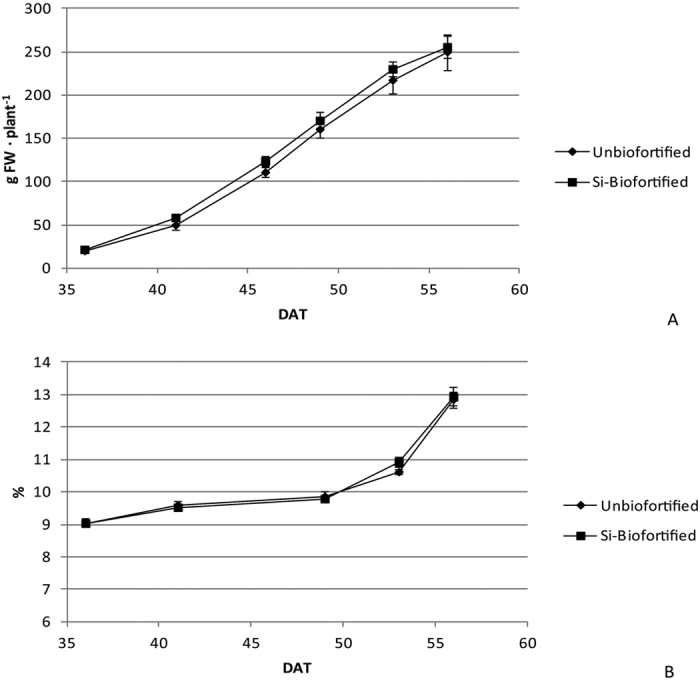
Cumulate yield (**A**) and trend of the pod dry matter percentage (**B**) during the harvesting period of soilless green bean subjected or non-subjected to Si-enriched nutrient solution (‘Biofortified’ and ‘Unbiofortified’, respectively).

No differences were observed between ‘Unbiofortified’ and ‘Biofortified’ treatments in terms of total yield (252 g∙plant^−1^, on average), total number of pods (91 per plant, on average), mean weight (2.7 g∙pod^−1^, on average) and mean dry matter percentage (10.4%, on average) of pods (data not shown). The cumulate yield and the evolution of the pod dry matter percentage, during the harvesting period of the green bean subjected or non-subjected to Si enrichment, are shown in Fig. 1. Yield at each single harvest was not affected by the treatment (Fig. 1A). Dry matter percentage showed an increasing trend, with values ranging from 9.0% at the beginning of the harvesting period to 12.9%, on average, at the end, without differences between “Unbiofortified’ and ‘Biofortified’ treatments. The dry matter increased more slightly during the first half of the harvesting period, while the increase became more pronounced during the second half (Fig. 1B).

### Si content in pods and other organs of plant

**Figure 2:**
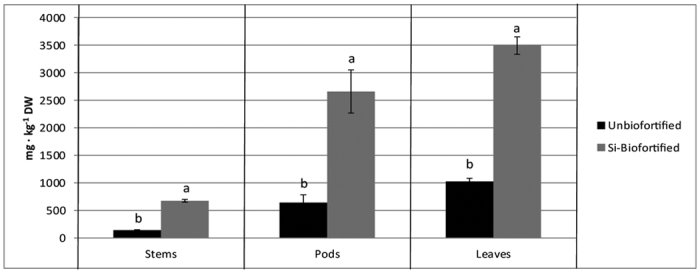
Si concentration in stem, pods and leaves of soilless green bean subjected or non-subjected to Si-enriched nutrient solution (‘Biofortified’ and ‘Unbiofortified’, respectively).

Aerial organs of plants subjected to Si enrichment showed an increased Si concentration if compared to plants irrigated with a NS without Si addition (Fig. 2). Si concentration increased approximately by 360%, 240% and 310%, on average, in stems, leaves and pods, respectively (Fig. 2). The addition of Si in the NS was effective in increasing steadily the Si concentration of the pods (Table 1). During the entire harvesting period, in fact, the Si concentration of the pods from the ‘Biofortified’ treatment was 192% higher, on average, than the ‘Unbiofortified’, without significant differences among the date of harvest (Table 1).

### Effects of cooking procedures on biofortified pods

Both the cooking procedures tested (steaming and boiling) did not affect the Si concentration in pods, which remained not different from that of raw pods. On average, the Si concentration of biofortified pods remained about 163% higher than unbiofortified, without differences among cooking procedures (Table 2). Si-biofortification did not affect on average color parameters, which were on the contrary significantly influenced by the cooking methods. In particular, L* and b* were higher in the raw pods (45.6 and 29.6, respectively) than in cooked ones (37 and 22 on average, respectively) without differences between the cooking methods (Table 2). Values of the other color parameters ranged from −16.9 to −6 for a*, from 119.8 to 105.6 for h°, and from 34.1 to 22.5 for C, respectively observed in raw and steamed pods, with intermediate values in boiled pods (Table 2). The Si bioaccessibility result was more than triple as a result of biofortification process, and was not affected by the cooking method (Table 2).

## Discussion

The first objective of the present study was to demonstrate the possibility to increase the Si concentration in green bean pods by acting on the growing technique, without affecting negatively the overall crop performance. Our results indicate that this goal was achieved by using soilless cultivation, with the use of perlite as a growing medium and supplying Si via the NS. The Si concentration in our biofortified green bean pods was on average about 3 times higher than the unbiofortified ones, and the effect was evident and stable during the entire harvesting cycle (Table 1). The crop performance was not influenced by the Si enrichment process in terms of yield and dry matter content of pods (Fig. 1). The biofortification was achieved by just modifying the composition of the NS with respect to a standard one and this modification consisting only in the addition of potassium metasilicate (K_2_SiO_3_). Under a practical point of view, the use of potassium metasilicate is to be preferred to other Si sources, because it prevents the occurrence of severe problems with blocking of the irrigation system occurred due to instability of other Si sources like oligomer silicic acid, and it is better absorbed by plants than Si colloids^30^.

In the present study, the Si concentration of unbiofortified pods resulted in line with the values reported for commercial green beans by Powell *et al*.^31^, who classify green bean among the richest sources of Si with respect to vegetables and legumes. In a previous Si biofortification study, D’Imperio *et al*.^6^ found significantly lower Si tissue content in 6 leafy vegetables, confirming the species-specific response to Si accumulation^7^,^32^. Thus, the technique used in this study was able to triplicate the Si content of a vegetable which is already considered a high-Si food.

Voogt and Sonneveld (2001)^30^ reported, in general, a Si content in tissue of plants grown in soilless conditions, significantly lower in comparison with horticultural crops grown in soil, as a result of limited space for root expansion and limited Si availability compared to soil. Those Authors classify perlite as a growing substrate with a very poor Si release to plants. We demonstrated that a proper Si fertilization in soilless conditions could not only overcome this problem, but also be a tool for the production of added-value green bean. In agreement with the present study, in a previous research conducted by the same team, soilless cultivation (floating system) was a successful tool for the Si biofortification of leafy vegetables^6^. In the present research, however, a different soilless system (cultivation on substrate instead of hydroculture), a different plant species and a different plant organ target (fruits instead of leaves) were adopted. In addition, the Si contents obtained in green bean (2496 mg Si ∙ kg^−1^ DW) are higher respect to those found in biofortified leafy vegetables^6^ (137–335 mg Si ∙ kg^−1^ DW, according to the species), confirming that Si content in plant tissues is related with species^7^ and that green bean is a good target for Si biofortification process.

Although the addition of Si in soilless conditions is generally associated to yield improvement, in the present study the yield was not influenced by the Si treatment. Benefits from enhanced Si concentration in the root environment, however, resulted highly dependent from the species, the growing conditions and the occurrence of stress conditions to plants. Among horticultural crops, cucumber, rose, zucchini and corn salad have been reported to benefit of Si supply in terms of yield, but for soilless green bean no yield differences have been found as a result of increased tissue Si content^18^,^19^,^30^,^33^,^34^. However, it should be outlined that in most studies the increased crop performance under Si treatment has to be related with an enhanced plant tolerance to abiotic or biotic stress conditions. The target of our process was the biofortification, not the improvement of the crop performance in terms of yield, and no stress conditions have been imposed to plants. In other reported research focused on biofortification for micronutrients through fertilization management approach, no yield differences were observed^6^,^35^,^36^.

However, although no specific tests have been conducted during the implementation of the present study, we found an increased Si content in all the aerial organs of the plant (namely stems, leaves and pods, Fig. 2). This may represent a potential benefit, in addition to biofortification, in terms of potential tolerance enhancement in biofortified green bean to stress conditions, occurring during the normal growing conditions^16^,^18^,^20^.

Since green bean is a vegetable typically consumed after cooking, another important objective of the present research was to verify that cooking process has no negative effects on biofortified product. Therefore, we tested the effects of two cooking methods (boiling and steaming) on Si content, Si bioaccessibility and visual quality of green bean pods. We chosen these two methods as the ones most widely used in home cooking as well as in the food industry for the production of frozen and ready-to-use vegetable products.

The Si concentration of biofortified pods remained significantly higher than unbiofortified also after cooking, without differences among cooking procedures (Table 2). It is well known that cooking process could reduce the content of elements in vegetables, especially in the case of boiling, due to solute losses into cooking water and particle leakage resulting from mechanical damage. Renna *et al*.^37^ found that boiling caused a reduction of sodium and potassium in chicory stems compared with steaming and uncooked samples. Wang *et al*.^38^ reported that cooking beans and chickpeas in boiling water resulted in a significant losses of potassium and magnesium. To our best knowledge, the literature lacks information with regard to the effect of cooking on silicon content in vegetables. At any rate in the present study both cooking methods had no negative effect in terms of silicon concentration in the pods. Therefore, it may be speculated that by applying the cooking treatments tested, the specific structural characteristics of the green bean tissues make silicon no susceptible to losses by leaching and/or leakage phenomena.

The beneficial effects of high Si intakes on human bone health has been demonstrated^8^,^9^,^10^. An intake of Si between 10 and 25 mg per day is recommended to achieve these beneficial effect^14^. Taking into account the mean Si concentration found in our biofortified pods and a mean dry matter value of about 11% at the cooking test (Table 2), an average portion of biofortified fresh green bean of about 76 g would guarantee an intake of 25 mg of Si, while about 200 g of unbiofortified green bean would be needed to obtain a Si intake at the same extent.

In order to better assess the potential beneficial effects of Si biofortified green bean, the bioaccessibility evaluation was performed. This parameter represents the percentage of Si released from the food matrix during the *in vitro* gastro-digestion process. Generally, the bioaccessibility of minerals in biofortified vegetables is influenced by the biofortification process. Similarly to our findings, the increase of the released Si as a result of biofortification has been demonstrated in previous studies by the same research group^6^. The bioaccessibility values in Si biofortified basil (23%) were similar to those found in green bean in the present research (Table 2). Other authors found similar results on zinc biofortified vegetables^39^.

We found that the Si bioaccessibility of biofortified green bean, subjected to boiling or steaming cooking according to the most common practice for this vegetable, was about three times higher compared to unbiofortified, without differences between the cooking methods (Table 2).

The cooking method can differently influence the mineral bioaccessibility, as reported in other studies on different mineral components, such as zinc and iron. The cooking methods tested in these previous researches (pressure cooking, regular pan, microwave cooking) were able to modify the bioaccessibility percentage, with contrasting effects in relation to the food matrix and the mineral nutrients investigated^40^,^41^. In particular, Hemelatha *et al*.^40^ report that Zinc bioaccessibility in *Phaseolus vulgaris* L. was reduced by pressure cooking but not by microwave cooking, while iron bioaccessibility was enhanced by pressure cooking and reduced by microwave cooking. The results discussed in the present paper showed that the cooking procedures tested (boiling and steaming) are not able to influence Si bioaccessibility in green bean.

The bioaccessibility assessment represents a fundamental step to investigate the potential beneficial effects of foods, and it is an important parameter to evaluate the nutritional efficiency of biofortified vegetables, by providing information on the percentage of absorption of a mineral^41^. Moreover, the bioaccessibility evaluation supports information related to the Si stability of biofortified green bean after gastro-intestinal condition, and could help in further *in vivo* studies on the bioavailability, that represents the fraction of a given compound or its metabolite able to reach the systemic circulation^42^.

**Figure 3:**
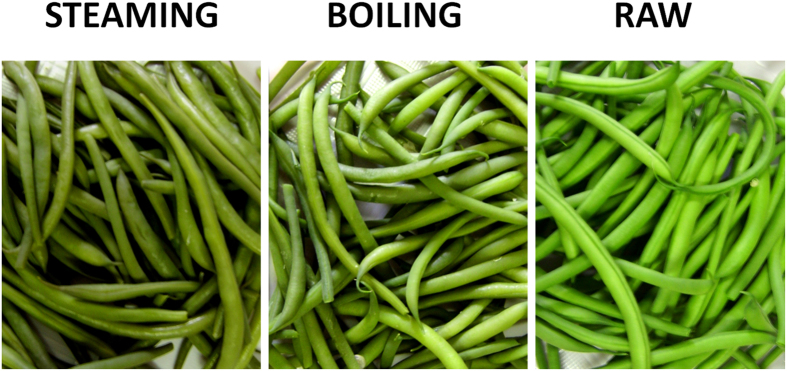
Silicon biofortified green bean pods uncooked or subjected to boiling or steaming cooking procedure.

Regarding the visual quality, the color of food surface is one of the main quality parameter evaluated by consumers, and it is critical to product acceptance. Food appearance, determined mostly by surface color, is the first sensation that the consumer perceives and uses as a tool to either accept or reject food^43^. Therefore, since color is an important quality attribute in the food industry and it influences consumer’s choice, a colorimetric analysis was performed. It focuses on the instrumental (objective) measurement for quantifying color attributes and highlights the range of primary and derived objective color indices used to characterize the quality of a wide range of food products. In the present study, color was not influenced by the biofortification process, while it was by cooking procedure, as expected (Table 2). Both cooking treatments caused an increase in redness (a^*^) and a decrease in lightness (L^*^) and yellowness (b^*^). These results are in agreement with Renna *et al*.^37^, who found similar changes in these color parameters in stem chicory using boiling and steaming. The differences between these color parameters are well represented by hue angle (h°) and chroma value (C) changes: a slight decrease was observed when boiling was used. These results agree with Turkmen *et al*.^44^ who found similar hue angle changes in green bean using boiling and steaming. These data indicated that boiling caused less color differences than steaming compared to uncooked green bean pods (Fig. 3). It is possible that these color differences in cooked pods could be attributed to a loss of chlorophylls and/or an increase in pheophytins and other chlorophyll derivatives^44^.

On the light of the above mentioned considerations, our findings about the possibility to increase on-field the Si content in green bean pods, and about the assessment of the Si bioaccessibility of the product ready for consumption (cooked), represent an important goal.

## Conclusions

In the present study we demonstrated that green bean is a good target for Si biofortification, since it can be biofortified for Si by growing plants in soilless conditions, using a Si-enriched nutrient solution. As a result, the final Si concentration of pods was almost tripled. The overall crop performance was not negatively influenced by the biofortification process, making this technique safe and easy to apply in commercial conditions. The Si content of biofortified pods was higher than unbiofortified ones also after cooking, no matter the cooking method used (boiling or steaming). Silicon bioaccessibility in cooked pods was more than tripled as a result of biofortification, independently of the cooking method used. Finally, biofortification process did not affect the visual quality of the product.

## Additional Information

**How to cite this article**: Montesano, F. F. *et al*. Green bean biofortification for Si through soilless cultivation: plant response and Si bioaccessibility in pods. *Sci. Rep.* **6**, 31662; doi: 10.1038/srep31662 (2016).

## Supplementary Material

Supplementary Information

## Figures and Tables

**Table 1:** Si concentration in pods of soilless green bean subjected or non-subjected to Si-enriched nutrient solution (‘Biofortified’ and ‘Unbiofortified’, respectively), at different dates during the harvesting period.

		Si concentration in the pods (mg Si ∙ kg^−1^ DW)
Si Biofortification	Unbiofortified	853.8
	Biofortified	2496.3
Date of harvest	36 DAT	1775.5
	41 DAT	1595.1
	56 DAT	1654.5
*Significance*^1^		
Si Biofortification		***
Date of harvest		ns
Si Biofortification * Date of harvest		ns

^1^Mean separation within columns by LSD_0.05_. ns and ***non significant or significant at P ≤ 0.001, respectively.

**Table 2:** Si concentration, color parameters (L*: lightness, a*: redness, b*: yellowness, h°: hue angle, C: saturation) and bioaccessibility (only for cooked material) in raw, boiled and steamed pods (harvested on 41 DAT) of soilless green bean subjected or non-subjected to Si-enriched nutrient solution (‘Biofortified’ and ‘Unbiofortified’, respectively).

		Si concentration(mg Si ∙ kg^−1^ DW)	L*	a*	b*	**h°**	**C**	Bioaccessibility%
Si Biofortification	Unbiofortified	1127.3	40.1	−10.9	24.4	113.1	26.9	7.6
	Biofortified	2972.4	39.7	−10.6	24.6	112.3	27.0	25.1
Cooking	Raw	1854.7	45.6 a	−16.9 a	29.6 a	119.8 a	34.1 a	—
	Boiling	2144.4	37.3 b	−9.3 b	22.3 b	112.7 b	24.2 b	15.7
	Steaming	2150.4	36.8 b	−6.0 c	21.7 b	105.6 c	22.5 c	16.9
*Significance*^1^								
Si Biofortification		***	ns	ns	ns	ns	ns	***
Cooking		ns	***	***	***	***	***	ns
Si Biofortification * Cooking		ns	ns	ns	ns	ns	ns	ns

^1^Mean separation within columns by LSD_0.05_. ns and ***non significant or significant at P  ≤  0.001, respectively.
