# Supplementary material for: Green bean biofortification for Si through soilless cultivation: plant response and Si bioaccessibility in pods
Source: Sci Rep. 2016 Aug 17;6:31662. doi: 10.1038/srep31662 (PMC4987696; doi:10.1038/srep31662)
Supplement: Supplementary Information [file srep31662-s1.doc]

**SUPPLEMENTARY INFORMATION**

**Green bean biofortification for Si through soilless cultivation: plant response and Si bioaccessibility in pods**

Francesco Fabiano Montesano^1^, Massimiliano D’Imperio^1^, Angelo Parente^1^, Angela Cardinali^1^, Massimiliano Renna^1,2^, Francesco Serio^1^

^1^Institute of Sciences of Food Production, CNR – National Research Council of Italy, Via G. Amendola, 122/O – 70126 Bari, Italy

^2^Department of Agricultural and Environmental Science – University of Bari Aldo Moro, Via Amendola, 165/A – 70126 Bari, Italy

The main information about the method (ASTM Method D589-00.27) and the related quality assurance parameters (QA), in relation to the typology of the sample analyzed (green bean pods or digested fluids) are reported in Table S1. The mean % of recovery of Si has been evaluated using the standard Na_2_SiO_3_ and a certified reference material.

**Tables S1. Quality assurance parameters of method used for Si analysis.**

|  | **Green bean** | **Digested fluid** |
| --- | --- | --- |
| Wavelength | 640 nm | 815 nm |
| Linear range | 0.40 – 5.68 mg · L^-1^ of Si | 352 – 1402 µg · L^-1^ of Si |
| Correlation Coefficient (R^2^) | 0.9997 | 0.9994 |
| Silicon standard (prepared in MilliQ H_2_O) | Na_2_SiO_3_ | Na_2_SiO_3_ |
| Certified reference material (CRM) | Silicon (Baker analyzed 5778-04) | Silicon (Baker analyzed 5778-04) |
| Quality assurance (QA) | | |
| Calibration verification standard (CVS %) | 1.78 | 4.65 |
| Mean % recovery of Si from Na_2_SiO_3_ (n = 4) | | |
| 2.81 mg·L^-1^ | 98.77±3.49 | - |
| 1.40 mg·L^-1^ | 95.27±1.78 | - |
| 0.748 mg·L^-1^ | - | 108.65±4.65 |
| Accuracy (Bias %) | -2.98 | 8.1 |
| LOD * | 0.11 mg·L^-1^ | 116.40 µg·L^-1^ |
| LOQ * | 0.34 mg·L^-1^ | 352.72 µg·L^-1^ |
| Mean % recovery of Si from CRM at different concentration (n= 5) from 0.75 to 5.00 mg·L^-1^) | 103.67±10.33 | - |
| Mean % recovery of Si from CRM at different concentration (n=5) from 400 to 1250 µg·L^-1^ | - | 96.34±7.76 |

*The limit of detection (LOD) and limit of quantification (LOQ) was calculated based on the standard deviation (SD) of the response and the slope (S) of the calibration curve at levels approximating the LOD and LOQ, LOD = 3.3 (SD/S) and LOQ = 10 (SD/S).
